# Compensation for the Public Services of Medical Men

**Published:** 1846-06-01

**Authors:** 


### *Compensation for the Public Services of Medical Men.*

The following extract from a communication by Dr. Ruschenberger, U. S. Navy, addressed to the Special Committee of the Assembly of New-York, in answer to inquiries concerning the subjects involved in the Quarantine Laws, embodies a fact which illustrates one aspect of the injustice of the public toward the medical profession. The subject deserves consideration, and we would suggest, in connection with it, for the reflection of our readers, the inquiry—in how far the profession itself is responsible for the custom which prevails with the public of availing itself *ad libitum* of its services without that equivalent which is regarded as indispensible when all other kinds of service are required :—*Ed. Buff. Jour.*

"There is a remark in your circular to which I beg leave to call your attention, because I conceive it bears strongly on the interests of the whole medical profession. You say, 'we are aware that a compliance with our request will impose considerable labor upon you, but we are persuaded that you will not hesitate to give our citizens the benefit of your learning and experience.' This is addressed to certain members of the medical profession, who are presumed by you to possess valuable information of a kind which would be a safe guide to enable you to draw such laws as would free commerce, in a measure, at least, from some of its restraints, without endangering the health of the community. You are aware, you state, that the expression of views and opinions which may have derived their sole value from many years of study 'will impose considerable labor,' and presume that this labor, learning and experience will be freely, gratuitously, given to our fellow citizens. I have no doubt that the result will prove your presumption to be correct; but is it either just, equitable, liberal or generous? Why should our fellow citizens expect medical men to serve the public gratuitously, while they freely and fully pay for the labors and services of every other class and description of men? In common with their fellow-citizens, medical men contribute their full quota in taxes, &c., to support the government; and as they enjoy no special exemptions, there seems to be no reason why they should pay extra in labor, &c., for the benefits of government. Lawyers and Legislators do not serve the people without pay. Attorneys and Judges are not expected, without remuneration, to protect society from the acts of murderers and felons by the administration of justice. Soldiers and Sailors are paid for defending the people from a common enemy in war. The Divine who addresses the the throne of grace in behalf of the representatives of the people assembled in the legislative hall, does not pray without reward. Why, then, should our fellow-citizens expect the gift of labor, time, learning and experience of a physician in cases where they are necessary for the common safety and benefit of society? Our fellow-citizens often require testimony which can be given only by a member of the medical profession, as in certain cases of inquest by coroners where the question of murder is involved; and it most frequently happens that while coroner, jurors, witnesses, attorneys, &c., are fully paid, the physician is merely thanked for his learning, experience and labor, without which they could not act." * * * *

"I trust I may be pardoned for my boldness in saying that, it seems to me unbecoming in the government of a great state to ask, through a committee of its legislature, gratuitous labor from individuals, and exclusively from one class of individuals."

